# Closing the Gap Between Need and Care: Predictors of Mental Health Service Use in EMS

**DOI:** 10.3390/ijerph23080978

**Published:** 2026-07-28

**Authors:** Kellie O’Dare, Clint Bowers, Deborah C. Beidel, Junwei Jiang, Steve McCoy

**Affiliations:** 1 UCF RESTORES, University of Central Florida, 4111 Pictor Lane, Suite 203, Orlando, FL 32816, USA; clint.bowers@ucf.edu (C.B.); deborah.beidel@ucf.edu (D.C.B.); 2 Florida Department of Health, Bureau of Emergency Medical Oversight, 4052 Bald Cypress Way, Bin A22, Tallahassee, FL 32399, USA; junwei.jiang@flhealth.gov (J.J.); steve.mccoy@flhealth.gov (S.M.)

**Keywords:** emergency medical services (EMS), mental health service utilization, help-seeking behavior, first responders, occupational mental health, peer support, behavioral health access, psychological distress, stigma, workforce health

## Abstract

**Highlights:**

**Public health relevance—How does this work relate to a public health issue?**

- EMS personnel represent a critical public health workforce; low utilization of mental health services in this population carries direct consequences for patient safety, workforce stability, and system capacity.
- Actual mental health service use—rather than intentions or attitudinal proxies—is the criterion in a statewide sample of 36,697 licensed Florida EMS professionals.

**Public health significance—Why is this work of significance to public health?**

- Psychological symptoms, not stigma, are the strongest factor associated with help-seeking, reframing how barriers to care are prioritized in high-risk occupational populations.
- Peer support is the most actionable system-level factor associated with increased service utilization, with meaningful organizational and workforce benefits.

**Public health implications—What are the key implications or messages for practitioners, policy makers and/or researchers in public health?**

- Findings are consistent with the potential value of routine symptom screening, proactive identification, and structured referral pathways within EMS agencies.
- Strengthening peer support infrastructure and confidential, occupationally competent clinical access offers a practical, scalable approach to improving workforce well-being and sustaining EMS system performance.

**Abstract:**

**Background:** Emergency medical services (EMS) personnel experience high rates of psychological distress yet demonstrate low utilization of mental health services. Factors associated with actual service use—rather than intentions or attitudes—are not well understood. **Methods:** Using secondary data from 36,697 licensed Florida EMTs and paramedics collected during the 2022 licensure renewal process, logistic regression models evaluated the relative contributions of workplace factors, attitudinal variables, trauma exposure, and mental health symptoms in their association with service utilization. **Results:** Psychological symptoms, particularly anxiety and depression, were the strongest factors associated with help-seeking. Peer support emerged as one of the few system-level factors associated with increased service use, even after accounting for symptom presence. Stigma-related variables showed weaker and, in some cases, counterintuitive associations. Suicidality and substance use were modestly associated with help-seeking beyond core symptom variables. Findings were consistent among trauma-exposed personnel. **Discussion:** Results are consistent with the potential value of early identification, routine symptom screening, and integrated, system-level supports such as peer support and access to confidential, occupationally competent care.

## 1. Introduction

The work of emergency medical services (EMS) professionals, including Emergency Medical Technicians (EMTs) and paramedics, is characterized by substantial physical workload and psychologically stressful conditions. Nonetheless, despite the growing availability of mental health services, EMS personnel report low rates of service use [1]. Florida alone licenses more than 77,000 EMTs and paramedics who collectively respond to hundreds of thousands of calls each year [2], a workforce whose mental health has been linked to outcomes for patient safety, crew functioning, and workforce stability [3]. Factors that influence actual mental health service use among EMS personnel, rather than simply intent or reported barriers to care, are not fully understood. The current study addresses this unknown regarding EMS professionals’ mental health service use by using actual service use as the criterion measure.

The occupational toll on EMS workers is well established. EMTs and paramedics are routinely exposed to scenes of serious injury, pediatric trauma, suicide, homicide, and mass casualty events [4,5]. The cumulative effect of these exposures is reflected in clinical data: rates of PTSD, major depression, and generalized anxiety disorder among EMS personnel consistently exceed those of the general population [6]. These psychological burdens do not arise from incident exposure alone. Shift schedules that disrupt sleep, sustained physical demands, and the emotional weight of patient care all contribute [7]. Therefore, a work environment is created in which psychological injury to the employee is not only likely but may also be inadequately recognized and addressed at the organizational level [8]. Low help-seeking persists even in agencies where mental health resources are provided. Factors have been identified that influence this behavior, including the culture of the EMS workplace, where “toughness” is a highly valued trait. Workers who express psychological difficulties are often stigmatized and perceived as not being able to perform the demands of the job [9]. But the barriers are not only cultural. EMS personnel face concrete professional consequences that workers in most other fields do not: fitness-for-duty evaluations, potential license jeopardy, and the judgment of colleagues who may interpret help-seeking as a liability [10]. In fact, many states condition licensure on medical fitness, and EMS personnel have legitimate concerns about the risk of licensure sanction if they engage in mental health treatment [11]. That uncertainty, even when the actual regulatory risk is low, is enough to suppress help-seeking.

Stigma is the most studied barrier to mental health care in first responder populations [8,10,12]. Research across police, fire, and military samples finds that both public stigma (anticipated judgment by others) and self-stigma (the internalized belief that needing help signals weakness) are significant barriers to seeking professional help [13,14]. Disclosure comfort, or the degree to which an individual feels comfortable talking about their mental health at work, is a third variable not fully explored in the context of first responders. Like stigma, disclosure comfort is likely a function of the workplace environment as much as an individual’s belief system. And similar to stigma, it is particularly impactful in environments like EMS, where there may be more social penalties to being seen as vulnerable [15].

In addition to attitudinal factors, symptom severity has consistently been linked to care-seeking [2,16]. Those with severe depression or anxiety symptoms are likely to experience not only suffering, but a greater likelihood of displaying their symptoms [17]. Peer interactions also warrant consideration. EMS crews typically work in small, close-knit units. Supportive peers may lower the threshold for disclosing distress and seeking care [18].

There are three specific gaps in the literature that we aim to fill in this investigation. First, attitudinal variables, symptom severity, trauma exposure, and workplace context variables have each been individually explored as correlates of seeking mental health services. However, no study has examined the relative impact of these variables in a sample of licensed EMS professionals who engage in actual help-seeking behavior (as opposed to intentions to seek such services). Second, the generalized EMS samples preclude examining the impact of stigma and symptom severity on help-seeking behavior in trauma-exposed providers, an especially relevant subgroup given possible atypical patterns of mental health service utilization. Third, for licensed EMS personnel, the impact of workplace-specific variables on help-seeking behavior remains unclear.

The purpose of this study is to address these gaps using survey data from a large cohort of licensed Florida EMS personnel (N = 36,697). The sample reported mental health symptoms, trauma exposure, attitudes towards providing care and work environment, and any subsequent utilization of available mental health or support services. Using incremental logistic regression, we examined the independent and relative contributions of attitudinal, symptom, trauma, and contextual factors in their association with actual mental health or support service utilization.

We repeated the core analyses in the subgroup of participants with trauma exposure to evaluate if findings were different for those who personally experienced trauma.

Our hypotheses were:

**H1.**  *Greater stigma (indicated by belief about being treated differently by peers and superiors), lower comfort disclosing mental issues, and lack of peer support are associated with lower odds of help-seeking.*

**H2.**  *Adding depression, anxiety, and sleep disturbance will be significantly associated with help-seeking above and beyond the base model.*

**H3.**  *Suicidality and substance use will show smaller incremental associations beyond core symptom variables.*

**H4** **(Trauma** **subgroup).** *Among trauma-exposed employees only, the pattern of associations will be more robust.*

## 2. Materials and Methods

This study utilized secondary data analysis of de-identified data obtained through a collaboration with the Florida Department of Health, Bureau of Emergency Medical Oversight. The study population consisted of licensed EMS professionals in Florida, including EMTs and paramedics, who completed the licensure renewal process during the study period (2022). As part of the biennial licensure renewal process required for all licensed EMTs and paramedics in Florida, a series of mental health and workplace-related items was incorporated into the electronic renewal questionnaire. All licensed EMS professionals in the state are required to complete this process every two years to maintain active licensure. The survey items were developed by Department of Health staff, with an emphasis on feasibility and brevity to allow inclusion within an otherwise lengthy licensure renewal process. Items were designed to capture self-reported mental health symptoms, trauma exposure, workplace supports, and service utilization while minimizing respondent burden. Respondents completed the licensure renewal questionnaire electronically through the Department of Health system. Following data collection, responses were de-identified by the Department of Health, and the dataset was transmitted to the research team for analysis.

The questionnaire included items assessing mental health symptoms, trauma exposure, substance use, and access to and perceptions of mental health services. Items were structured as self-report questions, measured as categorical (yes/no) responses. Mental health symptom items assessed experiences attributed to job demands, traumatic events, or work-related stress, including sleep disturbance, anxiety, depression, suicidal ideation, and substance use. Additional items assessed trauma exposure and use of mental health services outside of the employing agency. Workplace environment and organizational support items assessed the availability of behavioral health resources (e.g., Employee Assistance Programs, peer support, critical incident services), perceived accessibility of services, knowledge of how to access care, and perceptions of confidentiality, stigma, and potential career impact. Table 1 provides a list of the questionnaire items. Analytic procedures are described in Section 3. This study was reviewed and approved by the University of Central Florida Institutional Review Board (Study ID: 08900). We used three criteria to select variables to include in the logistic regression models: (1) theoretical relevance to help-seeking in occupational mental health as well as first responder help-seeking; (2) variables were available in the licensure renewal questionnaire; and (3) variables had sufficient endorsement rates so that stable estimates could be obtained in the regression models. As no information on the demographic variables of sex and age was collected as part of the licensure renewal process in a form that could be used as covariates in the regression models, these variables were not included in any of the regression models. These limitations of the data source are discussed in more detail in the Limitations section.

We also conducted a series of chi-square tests on included (n = 36,697) and excluded (n = 9107) cases on a set of demographic variables that were available for both groups. While the Cramer’s V values were small, indicating that the association between each variable and the included/excluded status was of little to no practical importance, there were several large absolute differences between included and excluded cases (i.e., cases that were kept in the analysis as well as those that were not). Specifically, 57.9% of the excluded cases held dual firefighter certification compared to 71.9% of the included cases, χ^2^(1) = 668.36, *p* < 0.001, Cramer’s V = 0.12. Thus, the complete-case sample appears to contain far fewer individuals with dual firefighter certification than the sample of all licensed EMS providers in Florida (a difference of approximately 14 percentage points). In addition, 7.3% of the excluded cases came from agencies located in rural areas compared to 3.4% of the included cases (a difference of a factor of 2.14 or approximately two-fold). Therefore, while help-seeking behavior among the complete-case sample of licensed Florida EMS providers may generalize to various subpopulations of providers, there are two groups of providers for whom help-seeking behavior may not generalize equally: (1) those with dual firefighter certification, and (2) EMS providers from rural agencies, for whom access to resources that could aid in dealing with a variety of job stressors may differ from that of their counterparts in non-rural areas as well as for whom occupation culture as a firefighter may differ from that of EMS providers in non-fire departments. This limitation is addressed in greater detail in Section 4.

## 3. Results

### 3.1. Preliminary Analyses

The initial dataset included 45,804 respondents. After excluding cases with missing data on help-seeking, perceived stigma, comfort with disclosure, trauma exposure, or peer support availability, the final complete-case sample for the logistic regression analysis comprised 36,697 EMS professionals. All continuous predictors were inspected for outliers and approximate linearity in the logit; no extreme outliers were removed. Categorical predictors were dummy-coded. The primary outcome was a binary indicator of service use. Overall, 6.0% of the complete-case sample (n = 2187 of 36,697) reported using mental health services. Table 2 presents the bivariate rate of service use by endorsement of each key predictor variable.

### 3.2. Base Model (Hypothesis 1)

A binary logistic regression analysis was conducted to evaluate whether stigma, comfort discussing mental health with supervisors, and peer support were associated with help-seeking behavior. Stigma was operationalized as endorsement of either peer-related or supervisor-related difficulty. Results indicated that respondents endorsing either form of difficulty had significantly higher odds of help-seeking than those endorsing neither. Peer support was also positively associated with help-seeking, such that respondents who reported peer support had higher odds of seeking help. In contrast, comfort discussing mental health concerns with a primary supervisor or command staff was not a statistically significant predictor in the multivariable model. Provider group remained a significant factor, with PMD respondents demonstrating greater odds of help-seeking than EMT respondents. Taken together, these findings provide partial support for the hypothesis. Specifically, peer support was associated with greater help-seeking, whereas the stigma indicator was associated with greater, rather than lower, odds of help-seeking. The base model demonstrated modest discrimination between help-seekers and non-help-seekers (AUC = 0.608). Confidence intervals for all odds ratios are presented in Table 3. The details of this analysis are presented in Table 3.

### 3.3. Incremental Value of Symptom Presence (H2)

To extend the first logistic regression analysis, anxiety and depression were added as factors to predict help-seeking behavior, alongside stigma, supervisor comfort, peer support, and provider group, so that the associations for all variables reflect effects adjusted for one another. In this model, both anxiety and depression were significant positive predictors of help-seeking. Anxiety was associated with increased odds of help-seeking, *B* = 0.947, OR = 2.58, *p* < 0.001, and depression was similarly associated with increased odds of help-seeking, *B* = 0.965, OR = 2.62, *p* < 0.001. As expected, the inclusion of anxiety and depression altered the magnitude of the coefficients for the social and organizational predictors, although these variables remained significant: stigma, *B* = 0.391, OR = 1.48, *p* < 0.001; supervisor comfort, *B* = 0.482, OR = 1.62, *p* < 0.001; and peer support, *B* = 0.564, OR = 1.76, *p* < 0.001. Provider group also remained a significant factor, with PMD respondents showing slightly higher odds of help-seeking than EMT respondents, *B* = 0.110, OR = 1.12, *p* = 0.016 (Table 4). Overall, the findings indicate that anxiety and depression are among the strongest correlates of help-seeking when considered jointly with other variables, while social and organizational factors continue to make independent contributions in the adjusted model.

### 3.4. Incremental Value of Suicidality and Substance Use (H3)

We next examined whether indicators of suicidality (Suicide) and alcohol/drug problems (AlcDrg) improved model fit beyond the base model and symptom severity. In the expanded model, all variables were significantly associated with help-seeking behavior (Table 5). Stigma remained a positive predictor of help-seeking, *B* = 0.230, *OR* = 1.26, *p* < 0.001. Supervisor comfort, *B* = 0.386, *OR* = 1.47, *p* < 0.001, and peer support, *B* = 0.585, *OR* = 1.80, *p* < 0.001, were also positively associated with help-seeking. PMD respondents continued to show slightly higher odds of help-seeking than EMT respondents, *B* = 0.109, *OR* = 1.12, *p* = 0.018. Among the clinical and behavioral health indicators, anxiety, *B* = 0.900, *OR* = 2.46, *p* < 0.001, depression, *B* = 0.733, *OR* = 2.08, *p* < 0.001, alcohol or drug concern, *B* = 0.379, *OR* = 1.46, *p* < 0.001, and suicidality, *B* = 0.738, *OR* = 2.09, *p* < 0.001, were all significant positive predictors. Overall, the findings indicate that anxiety and depression showed the largest associations with help-seeking when considered jointly with other variables (AOR = 2.58 and 2.61, respectively), while social and organizational factors continued to show independent associations in the adjusted model. Model discrimination improved substantially compared to the base model (AUC = 0.733 vs. 0.608).

### 3.5. Trauma-Exposed Subgroup Analyses (H4)

To test H4, we conducted formal interaction analyses in the full sample. For each predictor, a model was specified as: service use = predictor + trauma exposure + (predictor × trauma) + provider group. This approach provides a direct statistical test of whether the association between each predictor and help-seeking differs as a function of trauma exposure.

For stigma (B_interaction = −0.149, OR = 0.861, *p* = 0.228), comfort with disclosure (B_interaction = −0.101, OR = 0.904, *p* = 0.517), and all symptom-related predictors (anxiety, depression, sleep disturbance, suicidality, alcohol/drug concern; all *p* > 0.20), the predictor x trauma interactions were non-significant. This indicates that these associations did not differ significantly as a function of trauma exposure and supports the generalizability of the primary findings to trauma-exposed personnel. This analysis is presented in Table 6.

However, the peer support x trauma interaction was statistically significant (B_interaction = −0.513, OR = 0.599, 95% CI [0.388, 0.924], *p* = 0.019). Peer support was more strongly associated with help-seeking among personnel without trauma exposure (OR = 2.19) compared to those with trauma exposure (OR = 1.31). This finding indicates that the association between peer support and help-seeking is attenuated among trauma-exposed individuals relative to non-exposed individuals. These results are presented in Table 7.

Descriptive subgroup analyses in the trauma-exposed sample (n = 20,812; 8.7% service use rate; AUC = 0.581) are presented in Table 8 for reference. Taken together, formal interaction tests provide partial support for H4: findings generalized across trauma status for most predictors, with the notable exception of peer support.

## 4. Discussion

Prior investigations with first responders have typically examined stigma, workplace context, and psychological symptoms in isolation. Further, they have relied on intended rather than actual service use as the criterion. This investigation addressed both limitations in a statewide sample of licensed EMS professionals. The results suggest that the decision to seek mental health services is driven primarily by psychological symptoms rather than by the attitudinal and structural variables that have dominated prior theorizing. The data behind this conclusion, and its implications, are discussed below.

The first hypothesis, that greater stigma, lower comfort, and lack of peer support would be associated with lower odds of help-seeking, was only partially supported. Stigma, comfort with discussing mental health, and peer support were associated with help-seeking, but the base model by itself was not strongly predictive of that outcome. In the reverse direction, the positive association between stigma endorsement and help-seeking is somewhat counterintuitive. Several possible explanations for this finding should be considered. First, reverse causality is a possibility in a cross-sectional study such as this one: individuals who have already received treatment for their health problems may become more aware of stigma regarding mental health problems after their disclosure of needing treatment, compared with individuals who have not yet received treatment for their health problems. In addition, symptom confounding may account for part of the association between stigma endorsement and help-seeking in this study. That is, individuals with more severe health problems may seek help for their health problems for reasons mentioned above and report greater concern that they will receive less than equal treatment by other individuals compared with individuals with less severe health problems. In other words, in this study, the stigma item may function as a proxy for symptom severity in the base model. It is also possible that the single stigma item used in this study does not have adequate validity for measuring anticipatory stigma, which is the type of stigma that most theories consider to be a major barrier to help-seeking by first responders. Instead, the item may measure awareness of stigma that has already been experienced by an individual after disclosure of needing treatment for mental health problems. The item may also serve as an indicator of broader occupational distress or perceived workplace hostility. The association between stigma endorsement and help-seeking by first responders found in this study is likely to be more complex than a simple deterrent model and deserves further study, using a variety of validated stigma measures and a longitudinal design to attempt to disentangle the above explanations for this finding. Similarly, concerns about workplace treatment or occupational promotion were positively associated with service use when added to the base model, but the corresponding impacts were modest, suggesting they played only a small role in improving prediction accuracy.

In contrast, and consistent with Hypothesis 2, relatively small sets of psychological distress substantially enhanced the prediction of help-seeking beyond attitudinal and contextual factors alone. Suicidality and substance use (Hypothesis 3) were each significantly associated with help-seeking, but showed smaller incremental associations compared to anxiety and depression, as reflected in the marginal improvement in AUC between the H2 and H3 models (0.733 vs. 0.739). Whether these different outcomes are due to lower base rates of suicidality or of problematic substance use is unclear, but given the sample size, the difference is not attributable to insufficient statistical power. Furthermore, even when the sample was limited to only those individuals who reported exposure to trauma, the results of the larger sample were essentially replicated. The presence of anxiety, depression, or sleep disruption remained the factors most strongly associated with treatment-seeking, with peer support showing a smaller independent association. Model discrimination in the trauma-exposed subgroup was modest (AUC = 0.581), consistent with the base model performance in the full sample.

The fact that peer support was one of the few factors, outside of psychological symptomatology, that increased the likelihood of help-seeking is important. Departments and municipalities often question the need for peer support, citing legal, financial, and liability concerns. Nevertheless, given the high costs of early retirement, increased sick days, and disciplinary actions due to an employee’s emotional distress, even a small increase in help-seeking resulting from peer support could yield substantial (though as yet unmeasured) benefits.

A notable finding emerged from the interaction analyses conducted to formally test H4: the association between peer support and help-seeking was significantly moderated by trauma exposure (B = −0.513, OR = 0.599, *p* = 0.019). Peer support showed a stronger association with help-seeking among non-trauma-exposed personnel (OR = 2.19) than among trauma-exposed personnel (OR = 1.31). One interpretation is that trauma-exposed individuals may require more intensive or structured support interventions beyond informal peer contact to facilitate treatment engagement. Alternatively, trauma-related avoidance may attenuate the extent to which peer interaction translates into actual service use. This finding suggests that peer support programs may benefit from trauma-informed adaptations when targeting personnel with known trauma histories, and warrants replication with validated measures. benefits for departments.

Returning to the primary correlate, emotional distress, using a dichotomous variable (yes/no) may obscure potentially important findings regarding help-seeking behavior. Anxiety and depressive feelings, as well as sleep disruption, function along a continuum. The use of a dichotomous variable assigns everyone, regardless of severity, into the same category. Further understanding of help-seeking behaviors in EMS professionals may be better understood when psychological symptoms are assessed using a dimensional scale of symptom severity, rather than a simple presence/absence of emotional distress.

A related factor not assessed in the current investigation but often highly correlated with symptom severity is functional impairment. Specifically, it may not be severity alone, but the impact of emotional distress on occupational functioning, that is the final factor spurring someone toward help-seeking. For example, when depressive symptoms become so severe that work performance is affected, absences increase, and interpersonal functioning is impaired. Threats of disciplinary action and potential job loss may motivate someone to seek help. Further investigations should include measures of occupational functioning in order to better understand this relationship.

### Limitations

The measures used in the licensure renewal questionnaire were developed for administrative purposes and have not been previously used in formal measurement or validated by other research. In general, single items are insufficient for measuring symptom severity and duration, or for capturing change. The findings here have limited power to detect relationships between variables due to small-to-moderate effect sizes. For example, there may be a positive association between stigma endorsement and help-seeking for mental health and/or substance use problems because one item could be failing to distinguish between individuals’ anticipatory stigma concerns about mental health and/or substance use. Additionally, because this study was cross-sectional, it is not possible to make causal statements about the relationships found here. It may be the case that individuals who have sought help for mental health and/or substance use problems report greater numbers of symptoms and perceive more support from peers and available resources than others. In future studies, a longitudinal design can be used to explore reverse causality. Furthermore, all data for this study were collected via self-report, which has many types of potential reporting biases, especially for sensitive topics like suicidal ideation and substance use, in which there is a threat of mental health disclosure that could result in loss of licensure. Finally, the sample was from the state of Florida of licensed EMS workers. These findings here may not be generalizable to EMS workers in other states, to unlicensed EMS employees and other first-responder (including police and firefighters).

There are a few limitations to this study to note. For one, the complete-case analysis (CCA) of this study excluded 9107 or about 19.4% of the original sample of 46,714. Excluded certification categories were 57.9% dual compared to 71.9% dual in the included cases of 37,607. Additionally, agencies from rural settings made up 7.3% of all study participants as opposed to 3.4% from rural settings in the included cases of 37,607. Thus, findings from this study will not generalize to help-seeking behaviors of those in rural EMS agencies or those who are dual certified as firefighters.

A final limitation to highlight is that, although demographic information, for example, the sex and age of the respondent, is available in the dataset for all employees, this information is not available for the employees for whom data on licensure renewal is available. This variable is a well-established correlate of help-seeking behavior in a variety of occupations and therefore should be included as a covariate in the models, but is not available in this administrative dataset.

Note too that each of the single items used to measure the number of constructs within the administrative data source was used as ‘dichotomies’ (i.e., help-seeking for mental health problems by workers; help-seekers’ perceived stigma by others; and personal stigma by workers). It is possible that help-seeking for mental health problems by workers, for instance, is present but at a low level of severity and therefore of little consequence. Alternatively, it may be that the help-seeking is for problems of a minor nature only. Perceived stigma by others and personal stigma could also be based on anticipation as opposed to actual negative reactions by others. Finally, personal stigma could be based on negative beliefs that the worker holds about people with mental health problems in general and/or negative beliefs that the worker holds about having mental health problems himself or herself.

## 5. Conclusions

Findings suggest that improving mental health service utilization among EMS professionals may require moving beyond an exclusive focus on stigma reduction toward earlier identification, symptom recognition, and system-level supports. Symptom presence was a stronger predictor of help-seeking than attitudinal factors, although the use of a dichotomous variable precludes drawing conclusions about exactly how emotional distress increases the likelihood of help-seeking (whether the mere presence of negative affect or its severity leads to impairment). These findings are consistent with the potential value of routine symptom screening, structured referral pathways, and follow-up processes within EMS systems to support earlier engagement in care. Peer support also emerged as a meaningful factor associated with help-seeking. Even modest increases in utilization linked to peer support may have important organizational benefits, supporting its continued development as a core component of behavioral health infrastructure. Additionally, agencies should prioritize accessible, confidential, and occupationally competent clinical services, including clear communication about confidentiality and expanded access to vetted external providers.

Several priorities for future research emerge from these findings. First, validated measures of symptom severity and functional impairment are needed to clarify the threshold at which distress translates into help-seeking. Second, measuring workplace culture and stigma with validated scales rather than binary items would improve the precision of attitudinal variables. Third, longitudinal studies are needed to establish temporal order. Finally, studies that explicitly evaluate the impact of structured peer support programs and confidential referral pathways on utilization rates would advance the evidence base for system-level interventions.

## Figures and Tables

**Table 1:** Items assessing mental health symptoms, trauma exposure, substance use, and access to and perceptions of mental health services.

Survey Item	Response
Did your primary agency provide at least 2 h of continuing education regarding infectious diseases or ways to prevent transmission?	Yes/No
Experienced difficulty sleeping off duty attributed to job demands, trauma, or work stress	Yes/No
Experienced anxiety attributed to job demands, trauma, or work stress	Yes/No
Experienced depression attributed to job demands, trauma, or work stress	Yes/No
Had thoughts of suicide attributed to job demands, trauma, or work stress	Yes/No
Used alcohol or drugs attributed to job demands, trauma, or work stress	Yes/No
Experienced a traumatic event on the job	Yes/No
Accessed mental health services independently/outside agency	Yes/No
Agency offers Employee Assistance Program (EAP)	Yes/No
Agency offers peer support	Yes/No
Agency offers CISM/defusing/psychological first aid	Yes/No
Mental health support is readily available	Yes/No
Agency offers mental health training/education	Yes/No
Comfortable discussing mental health with supervisor	Yes/No
Believe supervisor would treat you differently if disclosed	Yes/No
Believe peers would treat you differently if disclosed	Yes/No
Believe disclosure would impact promotion	Yes/No
Believe agency mental health services are confidential	Yes/No
Know how to access mental health services	Yes/No
Agency offers chaplaincy services	Yes/No
Agency has health and safety committee	Yes/No
Accessed mental health services through agency (past 12 months)	Yes/No
If yes, satisfaction with services	7-point Likert scale

**Table 2:** Service Use by Predictor.

Variable	Endorsed n	Service Use (Endorsed)	Not Endorsed n	Service Use (Not Endorsed)	Difference
Stigma (boss or peer difficulty)	10,257	9.2%	26,440	4.7%	+4.5 pp
Comfortable disclosing to supervisor	28,556	5.7%	8141	6.9%	−1.2 pp
Peer support available	31,846	6.2%	4851	4.4%	+1.8 pp
Trauma exposure	20,812	8.7%	15,885	2.3%	+6.4 pp
Paramedic (vs. EMT)	15,758	6.9%	20,939	5.3%	+1.6 pp
Anxiety symptoms	12,386	11.9%	23,640	2.9%	+9.0 pp
Depression symptoms	7915	14.9%	28,111	3.5%	+11.4 pp
Sleep disturbance	13,191	11.1%	22,835	3.0%	+8.1 pp
Suicidal ideation	1265	27.3%	34,761	5.2%	+22.1 pp
Alcohol/drug concern	3603	17.7%	32,423	4.7%	+13.0 pp

**Table 3:** Base Model (H1)—N = 36,697, AUC = 0.608.

Variable	B	SE	OR	95% CI	*p*
Intercept	−3.360	0.066	0.035	[0.031, 0.039]	<0.001
Stigma	0.802	0.050	2.231	[2.020, 2.463]	<0.001
Comfort (supervisor)	0.087	0.059	1.091	[0.971, 1.226]	0.142
Peer support	0.520	0.079	1.681	[1.443, 1.959]	<0.001
Provider group (PMD)	0.253	0.044	1.288	[1.181, 1.405]	<0.001

Note. Outcome = help-seeking (1 = yes, 0 = no). ORs are adjusted odds ratios from a multivariable logistic regression model with all predictors entered simultaneously; 95% CI = 95% confidence interval. AUC = 0.608. Stigma operationalized as endorsement of either boss-related or peer-related differential treatment.

**Table 4:** Logistic Regression Predicting Help-Seeking: Base Model + Anxiety and Depression (H2).

Variable	B	SE	AOR	95% CI	*p*
Intercept	−3.979	0.072	0.019	[0.016, 0.022]	<0.001
Stigma	0.379	0.053	1.461	[1.316, 1.622]	<0.001
Comfort (supervisor)	0.465	0.061	1.592	[1.413, 1.794]	<0.001
Peer support	0.574	0.079	1.776	[1.522, 2.072]	<0.001
Provider group (PMD)	0.111	0.046	1.117	[1.021, 1.222]	0.016
Anxiety	0.948	0.064	2.580	[2.273, 2.929]	<0.001
Depression	0.959	0.062	2.609	[2.311, 2.946]	<0.001

Note. Outcome = help-seeking (1 = yes, 0 = no). AOR = adjusted odds ratio from a multivariable logistic regression model with all predictors entered simultaneously; 95% CI = 95% confidence interval. AUC = 0.733. Addition of anxiety and depression increased AUC by 0.125 over the base model (0.608), indicating a meaningful improvement in model discrimination.

**Table 5:** Logistic Regression Predicting Help-Seeking: Full Model with Suicidality and Substance Use (H3).

Variable	B	SE	AOR	95% CI	*p*
Intercept	−4.017	0.073	0.018	[0.016, 0.021]	<0.001
Stigma	0.223	0.055	1.249	[1.120, 1.393]	<0.001
Comfort (supervisor)	0.386	0.062	1.472	[1.304, 1.661]	<0.001
Peer support	0.581	0.080	1.788	[1.529, 2.091]	<0.001
Provider group (PMD)	0.109	0.046	1.115	[1.018, 1.221]	0.019
Anxiety	0.899	0.066	2.457	[2.159, 2.795]	<0.001
Depression	0.729	0.065	2.073	[1.819, 2.361]	<0.001
Suicidality	0.741	0.079	2.098	[1.796, 2.450]	<0.001
Alcohol/drug concern	0.384	0.062	1.468	[1.298, 1.661]	<0.001

Note. Outcome = help-seeking (1 = yes, 0 = no). AOR = adjusted odds ratio (all predictors entered simultaneously). CI = confidence interval. Full model AUC = 0.739, a marginal improvement over the H2 model (0.733), suggesting suicidality and substance use add limited discrimination beyond anxiety and depression.

**Table 6:** Predictor × Trauma Interaction Tests—Attitudinal/Contextual Predictors.

Predictor	B (Main)	OR (Main)	B (Trauma)	B (Interaction)	OR (Interaction)	*p* (Interaction)	Significant?
Stigma	0.656	1.928	1.338	−0.149	0.861	0.228	No
Comfort	0.058	1.060	1.458	−0.101	0.904	0.517	No
Peer support	0.785	2.192	1.840	−0.513	0.599	0.019	YES *

Note. * *p* < 0.05. Main effect for predictor = effect among non-trauma-exposed (trauma = 0). Interaction term = difference in the predictor effect between trauma-exposed and non-exposed. A negative interaction OR (<1) indicates that the predictor is less strongly associated with help-seeking among trauma-exposed individuals.

**Table 7:** Predictor × Trauma Interaction Tests—Symptom Predictors.

Predictor	B (Main)	OR (Main)	B (Trauma)	B (Interaction)	OR (Interaction)	*p* (Interaction)	Significant?
Anxiety	1.086	2.962	0.825	0.150	1.162	0.244	No
Depression	1.165	3.206	0.891	0.124	1.132	0.423	No
Sleep	1.085	2.960	0.948	−0.021	0.979	0.868	No
Suicidality	1.470	4.349	1.241	0.072	1.074	0.860	No
Alc/Drug	1.163	3.201	1.161	−0.062	0.940	0.803	No

Note. No symptom predictor × trauma interactions were statistically significant (all *p* > 0.20). This supports the interpretation that the association between symptom presence and help-seeking does not significantly differ as a function of trauma exposure status.

**Table 8:** Logistic Regression Predicting Help-Seeking: Trauma-Exposed Subgroup (H4).

Variable	B	SE	AOR	95% CI	*p*
Intercept	−3.274	0.084	0.038	[0.032, 0.044]	<0.001
Stigma	0.654	0.057	1.924	[1.721, 2.151]	<0.001
Comfort (supervisor)	0.235	0.065	1.265	[1.114, 1.435]	<0.001
Peer support	0.358	0.084	1.430	[1.213, 1.686]	<0.001
Provider group (PMD)	0.176	0.049	1.192	[1.082, 1.313]	<0.001

Note. Base model only (stigma, comfort, peer support, provider group) in the subset of respondents endorsing trauma exposure (n = 20,812). Approximately 8.7% of the subgroup reported service use. AUC = 0.581 in this subgroup. All predictors were significant. See Section 4 for formal interaction tests.

## Data Availability

The datasets presented in this article are not publicly available because they include EMS licensure data maintained by the Florida Department of Health. Requests to access the datasets should be directed to the Florida Department of Health, Bureau of Emergency Medical Oversight.

## References

[1] Kim J.E., Dager S.R., Jeong H.S., Ma J., Park S., Kim J., Choi Y., Lee S.L., Kang I., Ha E. (2018). Firefighters, Posttraumatic Stress Disorder, and Barriers to Treatment: Results from a Nationwide Total Population Survey. PLoS ONE.

[2] Florida Department of Health Emergency Medical Services. https://www.floridahealth.gov/licensing-regulations/emergency-medical-services-system/.

[3] Tikkanen V., Kääriäinen M., Roivainen P. (2025). The effect of ambulance clinicians’ well-being on occupational and patient safety in prehospital emergency medical services: A scoping review. Emerg. Crit. Care Med..

[4] Mishra S., Goebert D., Char E., Dukes P., Ahmed I. (2010). Trauma Exposure and Symptoms of Post-Traumatic Stress Disorder in Emergency Medical Services Personnel in Hawaii. Emerg. Med. J..

[5] Boland L.L., Kinzy T.G., Myers R.N., Fernstrom K.M., Kamrud J.W., Mink P.J., Stevens A.C. (2018). Burnout and Exposure to Critical Incidents in a Cohort of Emergency Medical Services Workers from Minnesota. West. J. Emerg. Med. Integr. Emerg. Care Popul. Health.

[6] Petrie K., Milligan-Saville J., Gayed A., Deady M., Phelps A., Dell L., Forbes D., Bryant R.A., Calvo R.A., Glozier N. (2018). Prevalence of PTSD and Common Mental Disorders amongst Ambulance Personnel: A Systematic Review and Meta-Analysis. Soc. Psychiatry Psychiatr. Epidemiol..

[7] Gonczaryk A., Chmielewski J., Strzelecka A., Fiks J., Witkowski G., Florek-Łuszczki M. (2022). Occupational Hazards in the Consciousness of the Paramedic in Emergency Medical Service. Disaster Emerg. Med. J..

[8] Edgelow M., Scholefield E., McPherson M., Legassick K., Novecosky J. (2022). Organizational factors and their impact on mental health in public safety organizations. Int. J. Environ. Res. Public Health.

[9] Lawn S., Roberts L., Willis E., Couzner L., Mohammadi L., Goble E. (2020). The Effects of Emergency Medical Service Work on the Psychological, Physical, and Social Well-Being of Ambulance Personnel: A Systematic Review of Qualitative Research. BMC Psychiatry.

[10] Johnson S., Wild J., Sanderson K., Kent B. (2022). Perceptions and Experiences of Mental Health Support for Ambulance Trust Employees. J. Paramed. Pract..

[11] Ntatamala I., Adams S. (2022). The Correlates of Post-Traumatic Stress Disorder in Ambulance Personnel and Barriers Faced in Accessing Care for Work-Related Stress. Int. J. Environ. Res. Public Health.

[12] Mackinnon K., Everett T., Holmes L., Smith E., Mills B. (2020). Risk of Psychological Distress, Pervasiveness of Stigma and Utilisation of Support Services: Exploring Paramedic Perceptions. Australas. J. Paramed..

[13] Grupe D.W. (2023). Mental Health Stigma and Help-Seeking Intentions in Police Employees. J. Community Saf. Well-Being.

[14] Krakauer R.L., Stelnicki A.M., Carleton R.N. (2020). Examining Mental Health Knowledge, Stigma, and Service Use Intentions Among Public Safety Personnel. Front. Psychol..

[15] Lyubykh Z., Turner N., Weinhardt J.M., Davis J., Dumaisnil A. (2025). Facilitating Mental Health Disclosure and Better Work Outcomes: The Role of Organizational Support for Disclosing Mental Health Concerns. Hum. Resour. Manag..

[16] Blais R.K., Hoerster K.D., Malte C., Hunt S., Jakupcak M. (2014). Unique PTSD Clusters Predict Intention to Seek Mental Health Care and Subsequent Utilization in US Veterans with PTSD Symptoms. J. Trauma. Stress.

[17] Rief W., Martin A., Klaiberg A., Brähler E. (2005). Specific Effects of Depression, Panic, and Somatic Symptoms on Illness Behavior. Biopsychosoc. Sci. Med..

[18] Gouweloos-Trines J., Tyler M.P., Giummarra M.J., Kassam-Adams N., Landolt M.A., Kleber R.J., Alisic E. (2017). Perceived Support at Work after Critical Incidents and Its Relation to Psychological Distress: A Survey among Prehospital Providers. Emerg. Med. J..

